# The importance of localizing pulmonary veins in atrial septal defect closure!

**DOI:** 10.1186/1749-8090-6-41

**Published:** 2011-03-30

**Authors:** Ahmad Ali Amirghofran, Ashkan Karimi, Gholam Hossein Ajami, Alireza Rasekhi

**Affiliations:** 1 Shiraz University of Medical Sciences, Division of Cardiovascular Surgery, Shiraz, Iran; 2 University of Florida, Division of Thoracic and Cardiovascular Surgery, Gainesville, FL, USA; 3 Shiraz University of Medical Sciences, Division of Pediatric Cardiology, Shiraz, Iran; 4 Shiraz University of Medical Sciences, Department of Radiology, Shiraz, Iran

## Abstract

An 8-year-old girl was admitted for a simple closure of echocardiographically diagnosed Atrial Septal Defect (ASD). During the operation the right pulmonary veins orifices were not detected in the left atrium and attempt to localize them led to the discovery of three additional anomalies, namely Interrupted Inferior Vena Cava (IIVC), Scimitar syndrome, and systemic arterial supply of the lung. Postoperatively these finding were confirmed by CT angiography. This case report emphasizes the need for adequate preoperative diagnosis and presents a very rare constellation of four congenital anomalies that to the best of our knowledge is not reported before.

## Background

The need for adequate preoperative diagnosis in the field of congenital heart surgery cannot be overemphasized. To this end many centers routinely use Intraoperative Trans-Esophageal Echocardiography (ITEE). Mayo clinic group in a study of 1002 congenital heart disease patients demonstrated that ITEE had major impact in 13.4% of cases defined as revealing any undetected pre or intaoperative information requiring an otherwise non-performed procedure during the surgery; however, the ASD secundum subset (67 cases) was the only primary diagnosis in this study that ITEE had zero major impact on and routine ITEE did not seem to be cost effective in this group[1]. In our experience also a comprehensive preoperative Trans-Thoracic Echocardiography (TTE) is considered adequate for delineation of simple cardiac defects such as ASD secundum unless the cardiologist is not satisfied with the quality of the study in which case preoperative TEE, cardiac MRI or ITEE is considered. In contrast to what was just mentioned the following case report serves as an example of inadequate TTE that failed to detect other major concomitant congenital heart defects accompanying an ASD secundum in an 8-year-old girl which had major impact on her operation.

## Case presentation

**Figure 1:**
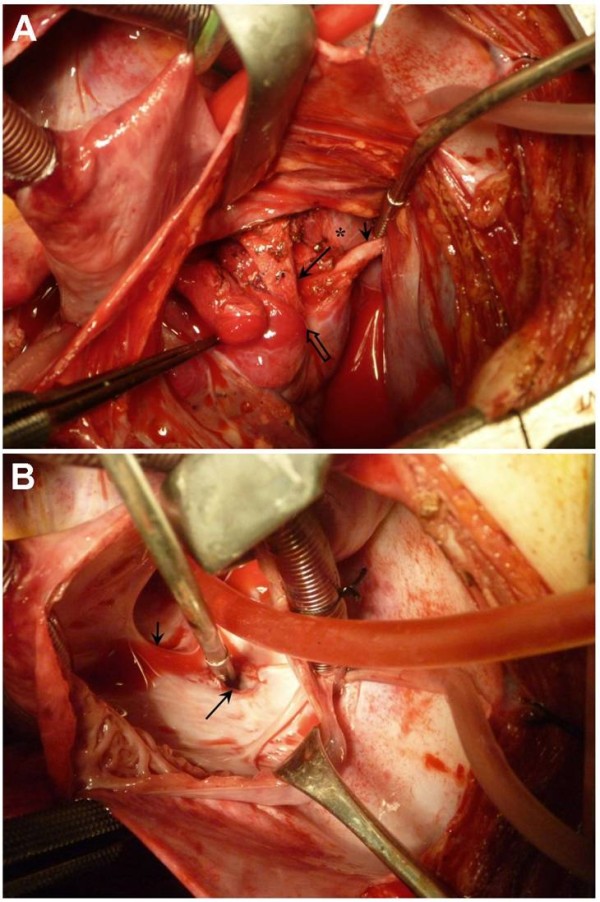
**Intraoperative view of the two anomalous blood vessels.** A - The long arrow shows the 12 mm vessel originating below the hilum of the right lung (hollow arrow) after being transferred to the right atrium. The small arrow shows the 7 mm vessel passing through the right dome of the diaphragm (*). B - Inside the right atrium is shown. The large arrow depicts the orifice of the redirected 12 mm vessel, which is fixed to the right atrium just at the right side of the ASD (small arrow). A pericardial patch is used later to redirect flow from this new orifice towards the left atrium

**Figure 2:**
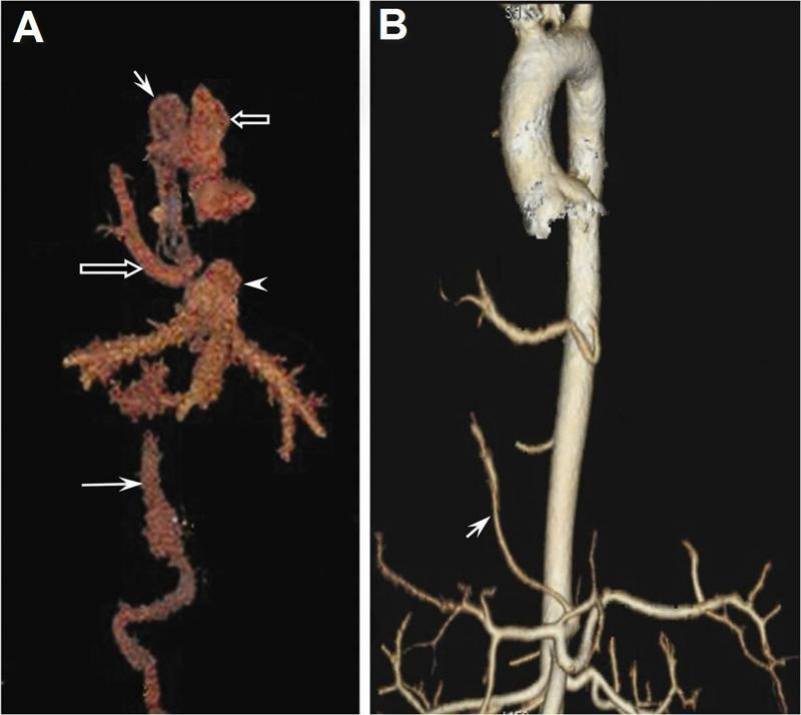
**A - Frontal projection of the venous phase of 3D CT angiography with volume rendering, which is obtained after the operation**. Annotated structures are: short solid arrow = enlarged azygos vein; long solid arrow = IVC, which is interrupted at the hepatic level; short hollow arrow = SVC; long hollow arrow = Redirected anomalous pulmonary vein; arrow head = hepatic vein, which drains into right atrium. B - The arterial phase depicts the anomalous systemic artery (arrow) arising from the celiac trunk and intending to supply the base of the right lung, which is ligated at the level of the diaphragm.

An 8-year-old girl referred with the complaint of mild exertional dyspnea. TTE revealed a 16 mm ASD secundum, moderate enlargement of the right atrium and ventricle, and an estimated Qp/Qs ratio of 2.3. Since the ASD did not have enough rim inferiorly to be closed by device, she was scheduled for surgical closure. Cardiopulmonary bypass was established by aorto-bicaval cannulation, and the ASD was approached through right atriotomy. Intraoperatively there was no trace of the right pulmonary veins orifices within either atria. Intrapericardial exploration of the distal Superior Vena Cava (SVC), as a common site for Partial Anomalous Pulmonary Venous Connection (PAPVC), failed to identify them and subsequently the right pleura was opened to look for them. Two vessels were discovered: a 12 mm vessel which ran between the right lung and the central part of the diaphragm and a 7 mm vessel which arose next to it and passed through the right dome of the diaphragm. Under the impression of Scimitar Syndrome, the Inferior Vena Cava (IVC) was decannulated to identify where these vessels drained into below the diaphragm. To our surprise, the IVC was interrupted bearing just few small orifices for the hepatic veins. Next the SVC was decannulated and dissected more superiorly to explore the enlarged Azygos vein which carries most of the subdiaphragmatic venous return to the heart in the setting of IIVC. Before transferring these two vessels as anomalous pulmonary veins to the left atrium we decided to confirm their drainage into the systemic venous circulation. A blood sample from the 12 mm vessel revealed 95% oxygen saturation and its baseline pressure was measured at 6-7 mmHg, then the Azyos vein was clamped immediately before its drainage into the SVC and the pressure readings gradually increased and established at 25 mmHg, implying that this vessel emptied into the systemic venous circulation under the diaphragm. Subsequently this vessel was cut at the level of the diaphragm to be transferred to the left atrium, but due to its short length we fixed it to the right atrium adjacent to the ASD in order to use an intraatrial baffle later for directing its flow to the left atrium (Figure 1). we were about to transfer the 7 mm vessel as another anomalous pulmonary vein that we noticed a considerable amount of bright red blood coming out from the site of new anastomosis, suggesting an aortopulmonary connection. A blood sample from the 7 mm vessel showed 97% oxygen saturation and upon its clamping the flow through the anastomosis stopped implying that the 7 mm vessel was a systematic artery which was supplying part of the right lung. Obviously the visible amount of shunt could not be left unattended; however, it was not clear whether this vessel was supplying normal lung tissue or an intralobar pulmonary sequestration. The patient had negative history for repeated pneumonia to suggest sequestration and the preoperative chest x-ray was normal. Given the small size of this vessel, we ultimately decided to ligate it without doing any resection and follow the patient closely after the operation in light of possible pulmonary necrosis and infection. In the end a pericardial patch was used to close the ASD and as an intraatrial baffle to direct flow from the anastomosed pulmonary vein to the left atrium. Fortunately the patient tolerated the procedure well and was discharged after 7 days without any pulmonary complication. Subsequent CT angiography 3 weeks later confirmed the intraoperative findings and showed homogenous lung parenchyma with no evidence of sequestration or necrosis (Figure 2).

## Discussion

This case report merits special consideration not only because of the very rare constellation of ASD, IIVC, Scimitar Syndrome, and anomalous systemic arterial supply of the lung, but also what the appropriate management should be while three of these four anomalies were discovered during the operation. It is not unheard-of for surgeons to come across new pathologies during the operation, but it is very unlikely for these new findings to change the nature of the procedure. In current practice TTE is considered adequate for the preoperative evaluation of ASD secundum and a detailed comprehensive echocardiography is expected to obviate the need for routine invasive diagnostic tests such as cardiac catheterization or even ITEE [1,2]. Preoperative TTE should include evaluation of all pulmonary veins. Occasionally they are not visualized due to poor acoustic window; in which case preoperative TEE, cardiac MRI or ITEE should be considered if not routinely performed [3]. Subcostal view in TTE should delineate Scimitar syndrome [3] and IIVC with Azygos continuation should also be readily diagnosed from this window [3]. Unfortunately these pathologies were missed in the preoperative TTE and were first diagnosed during the surgery. After IIVC was discovered intraoperatively the subject was raised to abort the operation and perform cardiac catheterization to accurately describe concomitant cardiac anomalies before proceeding further, but we ultimately decided to continue the operation and not to impose the risk of another surgery. Several surgical techniques are available to correct scimitar syndrome which are best summarized in the paper by Gudjonsson et al [4]. We applied the surgical technique introduced by Shumacker and Judd, which includes transfer of the anomalous pulmonary vein to the right atrium adjacent to the ASD and then baffling its flow across towards the left atrium [5]. Several other congenital anomalies are also reported in patients with IIVC including visceral heterotaxy and polysplenia which were absent in our case [6].

## Conclusions

Although preoperative TTE is considered adequate for the delineation of ASD secundum and its associated cardiac anomalies, this case report shows how an inadequate TTE can complicate the operation. Accordingly cardiologists should attempt to identify the site of drainage for all four pulmonary veins in the preoperative TTE and if there is any doubt about the quality of the study preoperative TEE, cardiac MRI or ITEE should be requested especially in centers where ITEE is not routinely performed for simple congenital heart surgeries such as ASD secundum closure.

## Consent

Written informed consent was obtained from the patient for publication of this case report and any accompanying images. A copy of the written consent is available for review by the Editor-in-Chief of this journal.

## Competing interests

The authors declare that they have no competing interests.

## Authors' contributions

AAA performed the surgery and supervised the manuscript. AK wrote the article and gathered the data. GHA contributed to the patient's care. AR interpreted radiographic images. All authors read and approved the final manuscript.
